# Maternal cariprazine exposure inhibits embryonic and postnatal brain cholesterol biosynthesis

**DOI:** 10.1038/s41380-020-0801-x

**Published:** 2020-06-05

**Authors:** Thiago C. Genaro-Mattos, Allison Anderson, Luke B. Allen, Keri A. Tallman, Ned A. Porter, Zeljka Korade, Károly Mirnics

**Affiliations:** 1 Munroe-Meyer Institute for Genetics and Rehabilitation, University of Nebraska Medical Center, Omaha, NE 68105 USA; 2 Department of Pediatrics, College of Medicine, University of Nebraska Medical Center, Omaha, NE 68198 USA; 3 Department of Chemistry, Vanderbilt University, Nashville, TN 37235 USA

**Keywords:** Biochemistry, Autism spectrum disorders

## Abstract

Cariprazine (CAR) is a strong inhibitor of the Dhcr7 enzyme, the last enzyme in the cholesterol biosynthesis pathway. We assessed the effects of CAR on maternally exposed *Dhcr7*^*+/−*^ and wild-type mouse offspring, and tested the biochemical effects of CAR in human serum samples. *Dhcr7*^*+/−*^ and wild-type time-pregnant mice were exposed to vehicle or 0.2 mg/kg CAR from E12 to E19. Levels of CAR, CAR metabolites, sterols, and oxysterols were measured in the brain of maternally exposed offspring at various time points using LC-MS/MS. Embryonic exposure to CAR significantly increased levels of 7-DHC in all organs of exposed embryos, with a particularly strong effect in the brain. Detectable levels of CAR and elevated 7-DHC were observed in the brain of newborn pups 14 days after drug exposure. In addition, CAR altered sterol metabolism in all animals analyzed, with the strongest effect on the brain of *Dhcr7*^*+/−*^ pups born to *Dhcr7*^*+/−*^ dams. Furthermore, CAR elevated toxic oxysterols in the brain of maternally exposed *Dhcr7*^*+/−*^ offspring to levels approaching those seen in a mouse model of Smith–Lemli–Opitz syndrome. Finally, we observed that patients taking CAR have elevated 7-DHC in their serum. In summary, maternal *DHCR7* heterozygosity, combined with offspring *DHCR7* heterozygosity might represent a vulnerability factor to medications that interfere with sterol biosynthesis. Due to the conserved sterol biosynthesis between mice and humans, we suggest that the 1–3% of patient population with single-allele *DHCR7* mutations might not be ideal candidates for CAR use, especially if they are nursing, pregnant or plan to become pregnant.

## Introduction

Cariprazine (CAR) (marketed under the name of Vraylar^®^) is a novel antipsychotic, approved in 2015 for the treatment of adults with schizophrenia and manic or mixed episodes associated with bipolar I disorder [1–3]. Based on Vraylar^®^ prescription information, CAR may cause fetal harm, and there are no adequate well-controlled studies in pregnant women [4]. However, animal studies suggest potential risks to a fetus, and patients should be properly advised [4].

Although its complete mechanism of action is still not fully understood, the primary effect of CAR is mediated through a combination of activity at D_2_ and D_3_ receptors and 5-HT_1A_ receptors [5, 6]. CAR is also an antagonist at 5-HT_2A_ and 5-HT_2B_ receptors and binds to the H_1_ receptors [7]. CAR major metabolites, desmethyl-CAR (DCAR), and didesmethyl-CAR (DDCAR) are pharmacologically equipotent to CAR [8]. DDCAR has been detected in the adult patients up to 12 weeks after discontinuation of CAR [4, 8]. In addition, CAR and aripiprazole (ARI), another atypical antipsychotic with a similar mechanism of action, are both metabolized to 2,3-(dichlorophenyl) piperazine (2,3-DCPP), which is an active and stable metabolite (Supplementary Fig. 1) [9–11].

In addition to the actions of the G-protein coupled receptors, our previous studies revealed that both CAR and ARI are strong inhibitors of the 7-dehydrocholestrol reductase (DHCR7), the last enzyme in the cholesterol biosynthesis pathway [10, 12–15]. The common metabolite of CAR and ARI, 2,3-DCPP, inhibits the DHCR7 activity at concentrations comparable to those of the potent teratogen AY9944 [10]. This DHCR7 inhibition by ARI, CAR, and AY9944 results in the elevation of 7-dehydrocholesterol (7-DHC), which is unstable and can give rise to toxic metabolites [16–18]. Previous studies from our group suggest that individuals with single-allele *DHCR7*^*+/−*^ mutations, who comprise ~1–3% of the human population, are particularly sensitive to 7-DHC elevating compounds, including ARI and trazodone [12, 13].

Knowing that cholesterol biosynthesis and cholesterol homeostasis are essential for the typical development of the brain, we sought to test the effects of CAR on the brain of maternally exposed offspring. We undertook a series of experiments in *Dhcr7*^*+/+*^ (WT) and heterozygous *Dhcr7*^*+/−*^ (Het) mice, analyzing levels of CAR and its metabolites in the brain of maternally exposed offspring. The obtained drug/metabolite data were correlated with levels of sterols and *Dhcr7* genotype. We also compared the levels of 7-DHC-derived oxysterols between CAR-exposed mice and a mouse model for Smith–Lemli–Opitz syndrome (SLOS). Finally, working with the Nebraska Biobank we were able to analyze the sterol content in human serum samples from individuals with CAR prescription and compare them to control individuals. The complete study design is outlined in Supplementary Fig. 2.

## Methods and materials

### Chemicals

Unless otherwise noted, all chemicals were purchased from Sigma-Aldrich Co (St. Louis, MO). HPLC grade solvents were purchased from Thermo Fisher Scientific Inc. (Waltham, MA). CAR was obtained from Sigma-Aldrich and dissolved in 0.9% saline solution for the experiments. All sterol standards, natural and isotopically labeled, used in this study are available from Kerafast, Inc. (Boston, MA).

### Mice studies

Full descriptions of the mice used in this study and the drug treatments performed are included in the Supplementary Material.

### LC-MS/MS (SRM) analyses

Sterols were analyzed as described previously [10]. A full description of the sterol analysis method is included in the Supplementary Material. CAR levels were acquired in an Acquity UPLC system coupled to a Thermo Scientific TSQ Quantis mass spectrometer using an ESI source in the positive ion mode. Five microliter of each sample was injected onto the column (Phenomenex Luna Omega C18, 1.6 μm, 100 Å, 2.1 × 50 mm) using water (0.1% v/v acetic acid) (solvent A) and acetonitrile (0.1% v/v acetic acid) (solvent B) as mobile phase. The gradient was: 10–40% B for 0.5 min; 40–95% B for 0.4 min; 95% B for 1.5 min; 95–10% B for 0.1 min; 10% B for 0.5 min. CAR and its metabolites were analyzed by selective reaction monitoring (SRM) using the following transitions: CAR 427 → 382, DCAR 413 → 382, DDCAR 399 → 382, and 2,3-DCPP 230 → 187. The SRM for the internal standard (d_8_-ARI) was set to 456 → 293 and response factors were determined to accurately determine the drug levels. Final drug levels are reported as ng/mg of protein.

### 7-DHC-derived oxysterol analysis

7-DHC-derived oxysterols (DHCEO, 4α-OH-7-DHC and 4β-OH-7-DHC) were analyzed by LC-MS/MS using an APCI source in the positive ion mode. Lipid content from 200 μL of brain lysate was extracted and the neutral lipids fraction was purified by SPE chromatography as described previously [19]. Purified content was resuspended in methanol and 5 μL was injected onto the column (Phenomenex Luna Omega C18, 1.6 μm, 100 Å, 2.1 × 100 mm) using ACN (0.1% v/v acetic acid) (solvent A) and methanol (0.1% v/v acetic acid) (solvent B) as mobile phase. The gradient was: 5% B for 2 min; 5–95% B for 0.1 min; 95% B for 1.5 min; 95–5% B for 0.1 min; 5% B for 0.5 min. The oxysterols were analyzed by SRM using the following transitions: DHCEO 399 → 381, 4α-OH-7-DHC 383 → 365, and 4β-OH-7-DHC 383 → 365. The SRM for the internal standard (D_7_-chol) was set to 376 → 376 and response factors were calculated to accurately determine the oxysterol levels. Final oxysterol levels are reported as nmol/mg of protein.

### Human serum analysis

Human serum and plasma samples were obtained from Nebraska Biobank: 27 serum or plasma samples with a CAR prescription listed in the medical records and 50 control serum samples (age, sex, and race matched). LC-MS/MS analyses were performed to detect and quantify CAR and its active metabolites in the 27 samples with CAR listed in the medical records. Out of twenty-seven, seven samples had detectable levels of CAR and its metabolites (two serum + five plasma samples). The absence of CAR and its metabolites in the remaining 20 samples can be due to several factors. It is possible, for instance, that the other 20 patients identified with CAR prescription were not taking the drug at the time (or close to the time) of the sample collection. Therefore, such samples were excluded from further analyses. To control for the difference between serum and plasma, seven blood samples were collected from control individuals, and serum and plasma were isolated from the same individual. The sterol profile was compared between serum and plasma and no difference was detected (data not shown). This comparison was conducted with an IRB approved protocol by UNMC.

### Statistical analyses

Statistical analyses were performed using Graphpad Prism 8 for Windows. Unpaired two-tailed *t*-tests were performed for individual comparisons between two groups. The Welch’s correction was employed when the variance between the two groups was significantly different. One-way ANOVA analyses were performed for comparisons between three or more groups. Two-way and three-way ANOVA analyses were performed to assess the interaction between maternal genotype, embryonic genotype, and drug treatment. The *p* values for statistically significant differences are highlighted in the figure legends.

## Results

### Maternal CAR exposure has a systemic effect on offspring with the most pronounced 7-DHC elevation in brain tissue

**Fig. 1:**
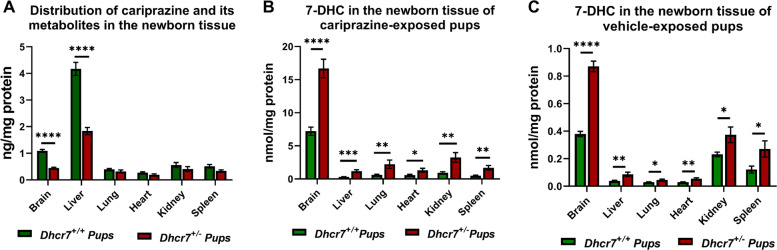
Maternal CAR exposure has a systemic effect on offspring with the most pronounced 7-DHC elevation in brain tissue. Samples were grouped taking into account the pups’ genotype and the levels of drugs were determined at P0. WT and *Dhcr7*-heterozygous pups are depicted in green or red, respectively. **a** Drug levels were determined in six different tissues and are presented as the sum of the parent drug and all active metabolites (CAR, DCAR, DDCAR, and 2,3-DCPP). **b** 7-DHC levels were measured across six different tissues of CAR-exposed pups and were found to be significantly higher in the *Dhcr7*^+/−^ pups when compared with their WT littermates. **c** Levels of 7-DHC in different tissues of WT and Dhcr7^+/−^ P0 pups after vehicle exposure; note that in all tissues measured values are significantly higher in the *Dhcr7*^+/−^ pups when compared with their WT littermates. The levels of 7-DHC in the CAR-exposed pups are significantly higher than in the vehicle-exposed group (note the different scale on the *Y*-axis). A different representation of this figure is shown in the Supplementary Fig. 3, denoting individual values and sex of the pups. Bars correspond to the mean ± SEM; statistical significance: *p < 0.05; **p < 0.01; ***p < 0.001; ****p < 0.0001.

To ascertain the biochemical consequences of maternal exposure to CAR on the brain of pups, timed-pregnant *Dhcr7*^*+/−*^ and WT mice received daily i/p injections of vehicle (VEH) or CAR (0.2 mg/kg) from E12 to E19. We measured the levels of CAR in six different organs in both WT and *Dhcr7*^+/−^ pups at P0. The drug amount is presented in Fig. 1a and it corresponds to the sum of CAR and its active metabolites (DCAR, DDCAR, and 2,3-DCPP). All compounds were detected in the brain, liver, lung, heart, kidney, and spleen. Out of those, the liver contained the highest levels of CAR and its metabolites, followed by the brain, kidney, lung, and spleen. These results suggest that CAR is distributed to multiple organs, systemically inhibits the Dhcr7 enzyme and increases 7-DHC across all tissues.

However, it appears that the most pronounced effect of CAR on 7-DHC was not in the liver, but in the brain tissue, by an order of magnitude (Fig. 1b). This suggests that the brain is particularly susceptible to DHCR7 inhibition. Furthermore, these data also revealed that pups with a *Dhcr7*^*+/−*^ genotype were more susceptible to DHCR7 inhibition (and consequent 7-DHC elevation) than their WT littermates. The magnitude of the 7-DHC increase in CAR-exposed pups was ~20-fold higher than the 7-DHC levels observed in VEH-injected animals (Fig. 1c**;** note the *y*-axis difference between Fig. 1b, c). It is also worth mentioning that no difference in the drug or 7-DHC levels was observed between male and female animals (Supplementary Fig. 3).

### CAR and its metabolites are detectable in the brain of maternally exposed offspring up to 14 days after birth

**Fig. 2:**
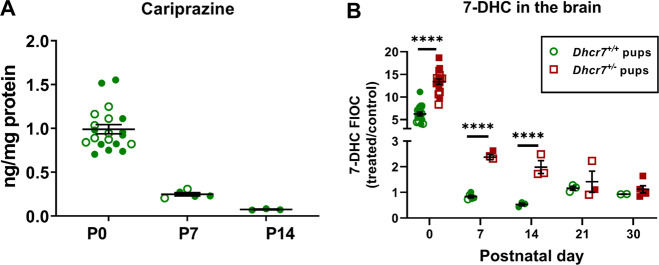
CAR and its active metabolites are detectable in the brain of maternally exposed offspring and results in long-lasting sterol changes. CAR **(a)** was quantified by LC-MS/MS in the brain of WT pups exposed to the drug in utero. Vehicle-treated control animals are not denoted in the figure, as they had no detectable CAR or metabolite levels. Levels of CAR-derived metabolites are depicted in Supplementary Fig. 4. **b** 7-DHC levels are presented as fold increases over control (FIOC: CAR-treated/SAL-treated pups) within each genotype over the course of 30 days after birth (at P0, P7, P14, P21, and P30). Each symbol corresponds to an individual pup brain; filled and opened symbols denote males and females, respectively; bars correspond to the mean ± SEM; statistical significance: ***p < 0.001; ****p < 0.0001. The chemical structures for cariprazine and its metabolites are depicted in Supplementary Fig. 1.

To further investigate the effects of maternal exposure to CAR on the brain of pups, we performed a time course analysis measuring the drug levels in the brain tissue in WT pups at P0, P7, and P14 (Fig. 2a). CAR was detected at all three postnatal time points, while its active metabolites (2,3-DCPP, DCAR, and DDCAR) were only detectable in all pups at P0 (Supplementary Fig. 4). In subsequent time points analyzed (P7 and P14), CAR metabolites were either detected in only part of the samples or not detected at all. The presence of CAR and its metabolites proves that CAR reaches the brain of the maternally exposed offspring, where it can elevate 7-DHC levels. Moreover, these results also suggest that the toxic 2,3-DCPP is the most abundant metabolite in the embryonic brain and that CAR effects may persist weeks after the drug has been discontinued.

To test the persistence of the CAR-induced biochemical changes in the pup brains over time, pregnant females were injected with CAR from E12 to E19 and 7-DHC levels in the brains of pups were assessed postnatally at P0, P7, P14, P21, and P30 (Fig. 2b). Figure 2b depicts changes in 7-DHC levels as fold changes over control, where values of CAR-exposed pups were divided by VEH-exposed pups within the same genotypes. The highest increase in 7-DHC was observed at P0 with *Dhcr7*-Het pups having significantly more 7-DHC than WT pups (WT: sixfold, *Dhcr7*^*+/−*^: 13-fold; *p* < 0.0001). In comparison with WT pup brains, 7-DHC stayed significantly elevated in *Dhcr7*^+/−^ pups through P14, gradually declining from P0. These data highlight the particular vulnerability the *Dhcr7*^*+/−*^ brain to CAR exposure even weeks after maternal CAR exposure has ended.

### Maternal exposure to CAR during pregnancy affects sterol profile in the brains of newborn pups in a Dhcr7 genotype-dependent manner

**Fig. 3:**
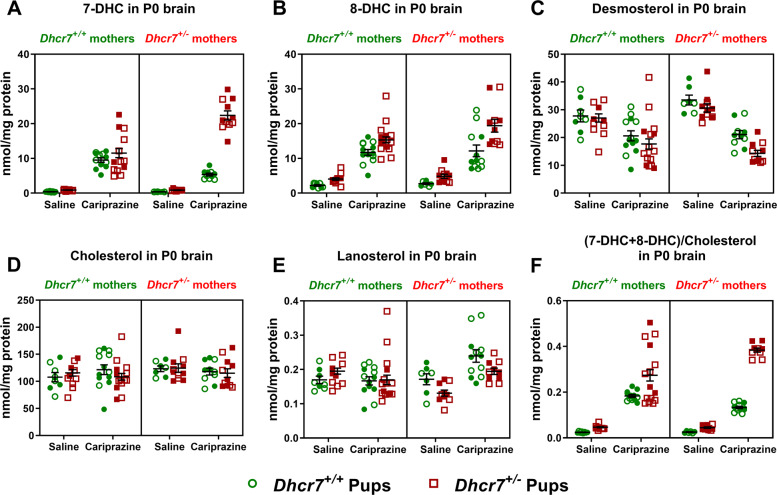
Maternal exposure to CAR during pregnancy affects sterol profile in the brains of newborn pups in a *Dhcr7* genotype-dependent manner. **a** Pregnant females were exposed to CAR from E12 to E19 and pups’ brains were analyzed for sterols at P0. Each symbol corresponds to an individual pup brain; filled and opened symbols denote males and females, respectively; bars correspond to the mean ± SEM. **a** 7-DHC; **b** 8-DHC; **c** desmosterol; **d** cholesterol; **e** lanosterol; **f** (7-DHC + 8-DHC)/cholesterol ratio. A comprehensive three-way ANOVA analysis of results is presented in Table 1.

Next, to gain a better understanding of the interaction between CAR, maternal *Dhcr7* genotype, and pup *Dhcr7* genotype on sterol levels, we analyzed by LC-MS/MS in eight groups of animals: (1) *Dhcr7*^*+/+*^ offspring of *Dhcr7*^*+/+*^ mothers; (2) *Dhcr7*^*+/−*^ offspring of *Dhcr7*^*+/+*^ mothers; (3) *Dhcr7*^*+/+*^ offspring of *Dhcr7*^*+/−*^ mothers; and (4) *Dhcr7*^*+/−*^ offspring of *Dhcr7*^*+/−*^ mothers, each treated with either VEH or CAR. A schematic representation of this experimental design is depicted in Supplementary Fig. 2A. Male and female animals are depicted in the figure as filled and opened symbols, respectively. These analyses revealed several important findings. First, maternal CAR treatment, regardless of maternal or offspring *Dhcr7* genotype, led to significantly elevated 7-DHC in all groups receiving treatment (Fig. 3a). Second, pups with a *Dhcr7*^*+/−*^ genotype were more vulnerable to CAR than their WT littermates. Third, brains from CAR-exposed *Dhcr7*^*+/−*^ offspring that were born from *Dhcr7*^*+/−*^ mothers showed the highest 7-DHC levels, with an ~26-fold increase in 7-DHC (*p* < 0.001) when compared with the VEH-exposed *Dhcr7*^*+/−*^ pups born from *Dhcr7*^*+/−*^ mothers. A similar effect was observed with 8-DHC, a compound that also increases when the DHCR7 enzyme is compromised (Fig. 3b). An analysis of the DHC/cholesterol ratio (calculated as (7-DHC + 8-DHC/cholesterol) further denotes the higher vulnerability of *Dhcr7*^*+/−*^ pups to CAR, especially those who were born from *Dhcr7*^*+/−*^ mothers (Fig. 3f). Similarly, CAR had a large effect on desmosterol levels (Fig. 3c). Desmosterol was strongly decreased in all maternally CAR-exposed pup brains, with a more robust effect observed in *Dhcr7*^*+/−*^ animals born from *Dhcr7*^*+/−*^ mothers (53% decrease from baseline, VEH-exposed *Dhcr7*^*+/−*^ pups, *p* < 0.001). In addition, the maternal *Dhcr7 heterozygosity* significantly contributed to the observed increase in 7-DHC and 8-DHC and the decrease in desmosterol. The combined increase in 7-DHC and 8-DHC with the decrease in desmosterol is consistent with the known outcome of the DHCR7 enzyme inhibition, as DHCR7 also converts 7-dehydrodesmosterol to desmosterol, thus resulting in a decreased enzyme product [10, 13]. In contrast, levels of cholesterol (Fig. 3d) and lanosterol (Fig. 3e) were only marginally changed in the brains of CAR-exposed P0 pups. No significant differences were observed between male and female pup brains. Table 1 depicts the statistical interaction between maternal *Dhcr7* genotype, pup *Dhcr7* genotype, and CAR treatment on 7-DHC, 8-DHC, desmosterol, and other sterol levels.

**Table 1:** ANOVA analysis of sterol levels in P0 brains.

#	Comparison	7-DHC	8-DHC	DES	CHOL	LAN
1	Treatment: VEH vs. CAR	**<0.0001**	**<0.0001**	**<0.0001**	0.7202	**0.0116**
2	Embryonic genotype: *Dhcr7*^*+/+*^ vs. *Dhcr7*^*+/−*^	**<0.0001**	**<0.0001**	**0.0157**	0.7655	0.1601
3	Maternal genotype: *Dhcr7*^*+/+*^ vs. *Dhcr7*^*+/−*^	**0.0121**	0.0630	0.2386	0.2235	0.3876
4	Two-way interaction: treatment (SAL or CAR) vs. embryonic *Dhcr7* genotype (^*+/+*^ or ^*+/−*^)	**<0.0001**	**0.0287**	0.2625	0.2654	0.5078
5	Two-way interaction: treatment (SAL or CAR) vs. maternal *Dhcr7* genotype (^*+/+*^ or ^*+/*^^−^)	**0.0110**	0.2975	**0.0244**	0.3507	**0.0001**
6	Two-way interaction: embryonic *Dhcr7* genotype (^*+/+*^ or ^*+/−*^) vs. maternal *Dhcr7* genotype (^*+/+*^ or ^*+/−*^)	**<0.0001**	0.2052	0.2711	0.8716	**0.0057**
7	Three-way interaction: treatment (SAL or CAR) vs. embryonic *Dhcr7* genotype (^*+/+*^ or ^*+/−*^) vs. maternal *Dhcr7* genotype (^*+/+*^ or ^*+/−*^)	**<0.0001**	0.2873	0.7525	0.4573	0.6526

Rows #1–3 denote statistical significance for single variables; #4–6 report probability for the two interacting factors; #7 reports probability three factors interacting; values highlighted in bold denote *p* < 0.05. No statistical difference was observed between male and female animals.

### Elevated 7-DHC levels result in the generation of toxic oxysterols at levels comparable to those seen in a Smith–Lemli–Opitz syndrome mouse model

**Fig. 4:**
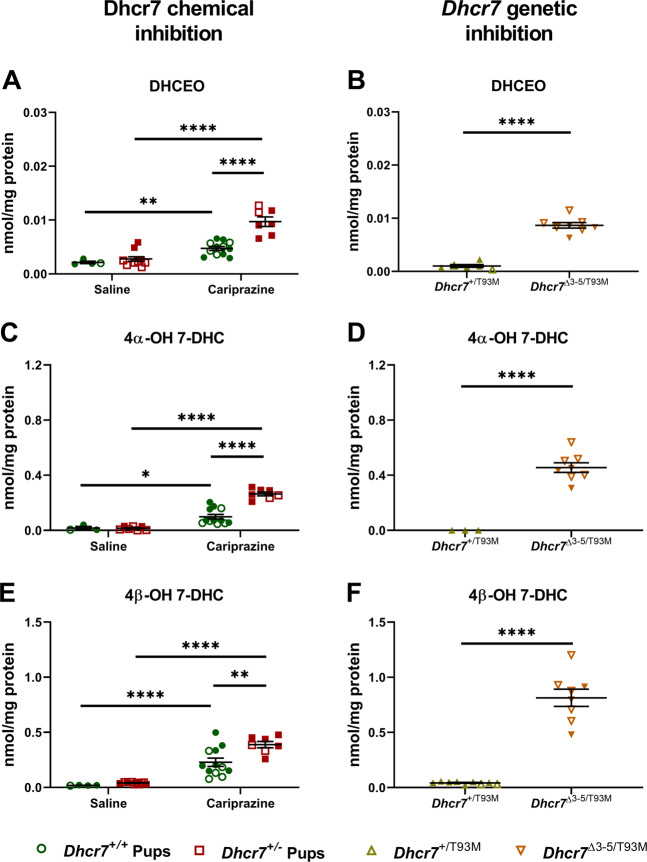
Elevated 7-DHC levels result in generation of toxic oxysterols at levels comparable to those seen in a Smith–Lemli–Opitz syndrome mouse model. Levels of DHCEO, 4α-OH-7-DHC, and 4β-OH-7-DHC were determined in the brains P0 pups. Samples were grouped taking into account the pups’ genotype. **a**, **c**, and **e** denote the comparison of the three oxysterols between SAL- and CAR-exposed pups. **b, d**, and **f** denote the comparison between *Dhcr7*^*+/T93M*^ (heterozygous) and *Dhcr7*^*Δ3-5/T93M*^ (compound heterozygous SLOS model) pups. Each symbol corresponds to an individual pup brain; filled and opened symbols denote males and females, respectively; bars correspond to the mean ± SEM; statistical significance: *p < 0.05; **p < 0.01; ****p < 0.0001.

7-DHC is the most oxidizable lipid known to date, and its toxicity may arise from its reactivity with peroxyl-free radicals and rapid oxysterol generation [18, 20, 21]. Previous experiments have shown that 7-DHC-derived oxysterols, such as DHCEO, have a huge effect on cell growth and differentiation [22]. In addition, DHCEO is considered a biomarker of increased oxidative stress in SLOS, an intellectual and developmental disability arising from two mutant copies of the *DHCR7* genes, biochemically characterized by elevation in 7-DHC [19, 23]. Thus, we assessed the formation of three 7-DHC-derived oxysterols (DHCEO, 4α-OH-7-DHC, and 4β-OH-7-DHC) in the brain of CAR-exposed pups. As hypothesized, all three oxysterols were significantly elevated in the brain tissue of P0 pups as a result of CAR exposure (Fig. 4a, c, e). Moreover, levels of these three oxysterols were significantly higher in the brain of *Dhcr7*^*+/−*^ pups, further highlighting their vulnerability to CAR treatment.

Next, we compared the CAR-induced oxysterol levels to those seen in the mouse model of SLOS (Fig. 4b, d, f). DHCEO levels were comparable between the *Dhcr7*^*Δ3-5/T93M*^ (compound heterozygous SLOS mouse model) pups and the CAR-exposed *Dhcr7*^*+/−*^ pups (CAR-exposed *Dhcr7*^*+/−*^ pups: 0.005 ± 0.00036 nmol/mg of protein vs. SLOS *Dhcr7*^*Δ3-5/T93M*^ pups: 0.009 ± 0.00056 nmol/mg of protein, *p* = 0.77) (Fig. 4a, b), while 4α-OH-7-DHC levels resulting from maternal CAR exposure by *Dhcr7*^*+/−*^ pups reached 60% of those seen in the SLOS mouse model (Fig. 4c, d). Similarly, 4β-OH-7-DHC levels of CAR-exposed in *Dhcr7*^*+/−*^ pups were 48% of those seen in the *Dhcr7*^*Δ3-5/T93M*^ pups (Fig. 4e, f). No sex differences were observed in oxysterol generation. These results suggest that CAR inhibition of *Dhcr7* is a potentially meaningful biological event that could significantly alter newborn brain development, especially in combination with a *Dhcr7*^*+/−*^ genotype.

### CAR elevates circulating 7-DHC levels in humans

**Fig. 5:**
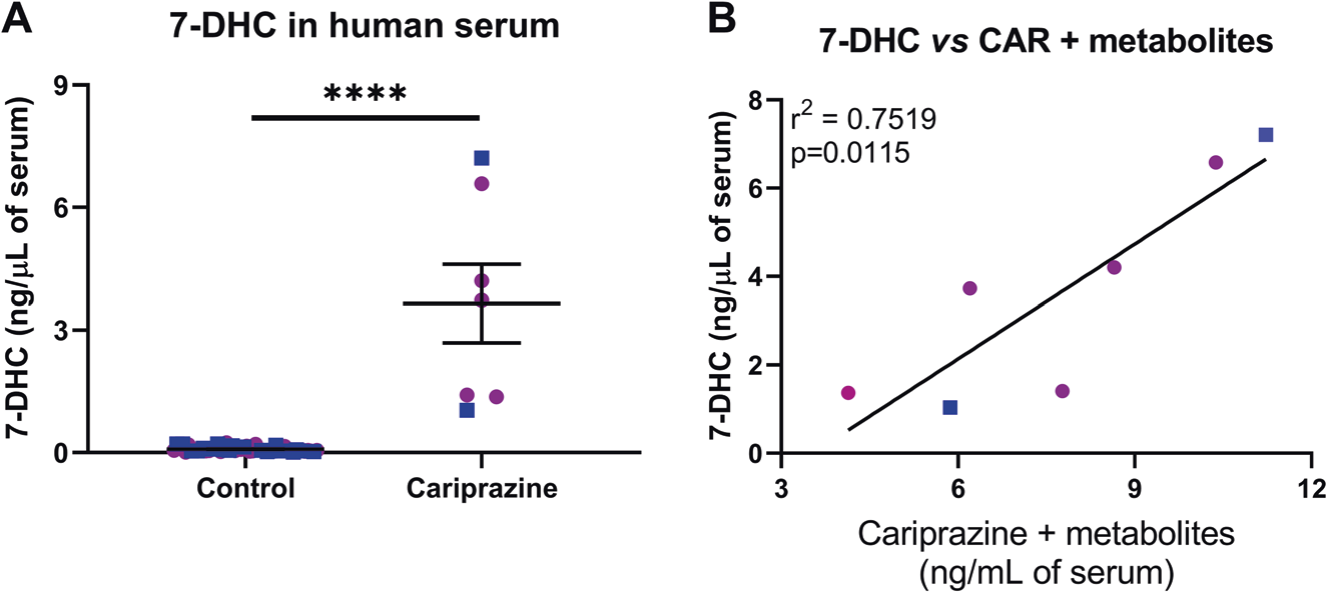
CAR elevates circulating 7-DHC levels in human serum. **a** 7-DHC is significantly elevated in seven human serum samples with detectable levels of CAR and its active metabolites when compared with control samples. Control samples originate from 50 age and sex-matched individuals with no prescription of CAR and no detectable levels of the active metabolites. Bars correspond to the mean ± SEM; statistical significance: ****p < 0.0001. **b** Correlation of 7-DHC and the drug levels detected in each human serum sample. Drug levels correspond to the sum of CAR and its active metabolites (DCAR, DDCAR, and 2,3-DCPP). 7-DHC is significantly correlated with drug concentrations (*r*^2^ = 0.7519; *p* = 0.0115). Blue and purple symbols denote male and female individuals, respectively. Information about age and sex of the donors can be found in Supplementary Table 1.

Our previous study showed that individuals taking ARI have significantly increased circulating levels of 7-DHC [15]. Since ARI and CAR have 2,3-DCPP as a common metabolite, we determined if taking CAR would increase circulating 7-DHC, as we observed with ARI. Twenty-seven serum samples from the Nebraska Biobank (with CAR prescriptions listed in their de-identified medical record) were tested for the presence of CAR and its metabolites. Of these, only seven samples had measurable amounts of CAR and its metabolites. These seven samples also had greatly elevated 7-DHC, when compared with 7-DHC levels measured in 50 age- and sex-matched control samples without detectable CAR levels in the serum (Fig. 5a). Furthermore, there was a strong correlation between CAR + metabolites serum levels and 7-DHC levels (*r*^2^ = 0.75; *p* = 0.01; Fig. 5b). These results suggest that the effects of CAR are similar in mouse in vivo models and the human population.

## Discussion

The main conclusions of our study can be summarized as follows: (1) in a rodent model, CAR treatment during pregnancy crosses the placenta and can be detected in multiple tissues of the offspring. (2) Embryonic exposure to CAR significantly increases levels of 7-DHC in all organs of exposed embryos, with a particularly strong effect in the brain of exposed pups. (3) CAR and its active metabolites are detectable in the tissue of newborn pups for at least an additional 14 days after the drug exposure has ended. (4) 7-DHC levels remain elevated in the pup brains for about the same time as CAR and its metabolites are detectable. (5) CAR alters sterol metabolism in all animals analyzed, but maternally exposed pups with *Dhcr7*^*+/−*^ genotype are more vulnerable than their WT littermates. (6) The combination of *Dhcr7*^*+/−*^ maternal and *Dhcr7*^*+/−*^ embryonic genotypes is the most affected group by CAR exposure. (7) CAR elevates toxic 7-DHC-derived oxysterols in the brain of maternally exposed offspring, in particular in animals with a *Dhcr7*^*+/−*^ genotype. (8) Patients taking CAR have elevated circulating 7-DHC in their serum.

The detection of CAR and its metabolites postnatally (up to 14 days) in the offspring brain is of particular significance. At this stage of life, there are critical processes that require sterols occurring in the developing brain [24]. For instance, rodent postnatal day (PND) 1–3, which corresponds to human 23–32 week gestation (pre-term infant), is characterized by oligodendrocyte maturation changes and predominance of mitotically active pre-oligodendrocytes as well as immune system development [25–27]. Rodent PND 7–10, equivalent to human 36–40 week gestation (term infant), is characterized by peak brain growth spurt, peak in gliogenesis, increasing axonal and dendritic density, consolidation of the immune system, and oligodendrocyte maturation [25, 27–29].

Cholesterol does not cross the blood–brain barrier, and cholesterol obtained from the diet or synthesized by the liver and various somatic cells does not contribute to the brain cholesterol pool. Yet, the brain is the most lipid-rich organ: it contains 25% of all lipids in the human body, and cholesterol makes up ~25% of the lipids in the brain [30, 31]. Thus, the brain synthesizes its own cholesterol, with both neurons and glia contributing to the brain sterol pool [31, 32]. As cholesterol in the brain serves as a critical precursor and structural building material, genetic disruptions in sterol biosynthesis result in a host of intellectual and developmental disabilities [33–37].

Disruption of DHCR7, the last enzyme of the cholesterol biosynthesis pathway, is perhaps the most interesting in this regard. Two copies of mutant *DHCR7* genes give rise to SLOS, a developmental disability characterized by elevated 7-DHC levels. 7-DHC is the most oxidizable lipid known to date, spontaneously giving rise to dozens of toxic oxysterols that adduct to proteins and disrupt differentiation and development [16, 18, 20–22, 38, 39]. Thus, our findings that CAR-induced 7-DHC levels and the increased DHCEO levels detected in *Dhcr7*^*+/−*^ pups are in the range of that seen in SLOS mouse models (genetic vs. chemical inhibition) are noteworthy. Importantly, the presence of a maternal *Dhcr7* genotype is also of critical importance in this regard: CAR elevation of 7-DHC is significantly higher in the pups of *Dhcr7*^*+/−*^ mothers than those of WT. The effect of CAR is best explained by the maternal Dhcr7 genotype*offspring Dhcr7 genotype*CAR treatment model.

There are potentially important clinical implications of our studies. CAR and ARI have similar pharmacological properties affecting, to different degrees, dopaminergic and serotonergic receptors. They also have similar substructural elements, and favorable clinical outcomes [10]. However, due to their related structure, they also share a common side effect, the inhibition of the DHCR7 enzyme, the last step in cholesterol synthesis. Consequently, due to the high oxidizability of 7-DHC, 7-DHC-derived oxysterols are formed and have detrimental effects on cellular development [16, 18, 20]. As the sterol synthesis pathway is conserved between mice and humans, and as we observed 7-DHC elevation in the patients who take CAR, we believe that our animal studies are highly informative and cautionary about the use of CAR in human pregnancy, especially in cases when either the unborn child or the mother is of the *DHCR7*^*+/−*^ genotype. It is also worth noting that primates and rodents have the same hemochorial type of placenta. It has been reported that the interspecies differences in the mammalian placenta do not play a dominant role in the placental transfer of most drugs, which is determined largely by placental blood flow [40]. Importantly, CAR (and its metabolites) can be detected in the blood of patients 3 weeks to 3 months after the drug treatment has been terminated [4], and this property may be less desirable when female patients who carry *DHCR7* mutations are pregnant or plan to become pregnant. Furthermore, individuals with the *DHCR7*^*+/−*^ genotype may want to limit the use of pharmaceuticals that act as DHCR7 inhibitors (including trazodone, ARI, and CAR), as both brain and systemic effects might arise. The significantly higher levels of these toxic oxysterols detected in the CAR-exposed pups (that are comparable to those seen in SLOS) suggest that oxidative stress is increased in these animals, and this warrants further studies.

It is also noteworthy that CAR and ARI are not the only two known inhibitors of DHCR7 [13, 14, 41]. A side effect of elevated 7-DHC levels have been described for a number of commonly prescribed pharmaceutical compounds, including trazodone, haloperidol, sertraline, amiodarone, trifluoperazine, fluphenazine, perphenazine [14, 15, 41, 42], and various chemicals in our surroundings [43–45]. Undoubtedly, while they are safe and effective compounds for the vast majority of human population, we believe that their effects in the context of patient genotype and life condition (e.g., pregnancy or comorbidity) remains understudied.

The utility of CAR to treat schizophrenia and bipolar disorder in the general population is not questioned by our findings. However, in the light of our results, it is important to consider alternative medications for use during pregnancy. From a precision medicine standpoint, one should consider that the 1–3% of patient population with single-allele *DHCR7* mutations might not be the ideal candidates for use of CAR, especially if they are nursing, pregnant, or plan to become pregnant.

## Supplementary information

Supplemental material
