# Supplementary material for: Maternal cariprazine exposure inhibits embryonic and postnatal brain cholesterol biosynthesis
Source: Mol Psychiatry. 2020 Jun 5;25(11):2685–94. doi: 10.1038/s41380-020-0801-x (PMC7577905; doi:10.1038/s41380-020-0801-x)
Supplement: Supplementary file 1 — Supplemental material [file 41380_2020_801_MOESM1_ESM.docx]

**Maternal Cariprazine Exposure Inhibits Embryonic and Postnatal Brain Cholesterol Biosynthesis**

*Thiago C. Genaro-Mattos^1^, Allison Anderson^1^, Luke B. Allen^2^, Keri A. Tallman^3^, Ned A. Porter^3^, Zeljka Korade^2*^ and Károly Mirnics^1*^*

^1^Munroe-Meyer Institute for Genetics and Rehabilitation, University of Nebraska Medical Center, Omaha, NE, USA, 68105.

^2^Department of Pediatrics, College of Medicine, University of Nebraska Medical Center, Omaha, NE, USA, 68198.

^3^Department of Chemistry, Vanderbilt University, Nashville, TN, USA, 37235.

*Supplemental Figure 1*…………………………………………………….………………Page S2

*Supplemental Figure 2*…………………………………………………….………………Page S3

*Supplemental Figure 3*…………………………………………………….………………Page S4

*Supplemental Figure 4*…………………………………………………….………………Page S5

*Supplemental Table 1*…………………………………………………….…………..……Page S6

*Methods………………*…………………………………………………….………….…Pages S7-8

*References…………*…………………………………………………….……………....…Pages S9

**Supplemental Figure 1**





**Supplemental Figure 1. Chemical structures of CAR into DCAR, DDCAR and 2,3-DCPP.**

**Supplemental Figure 2**

**
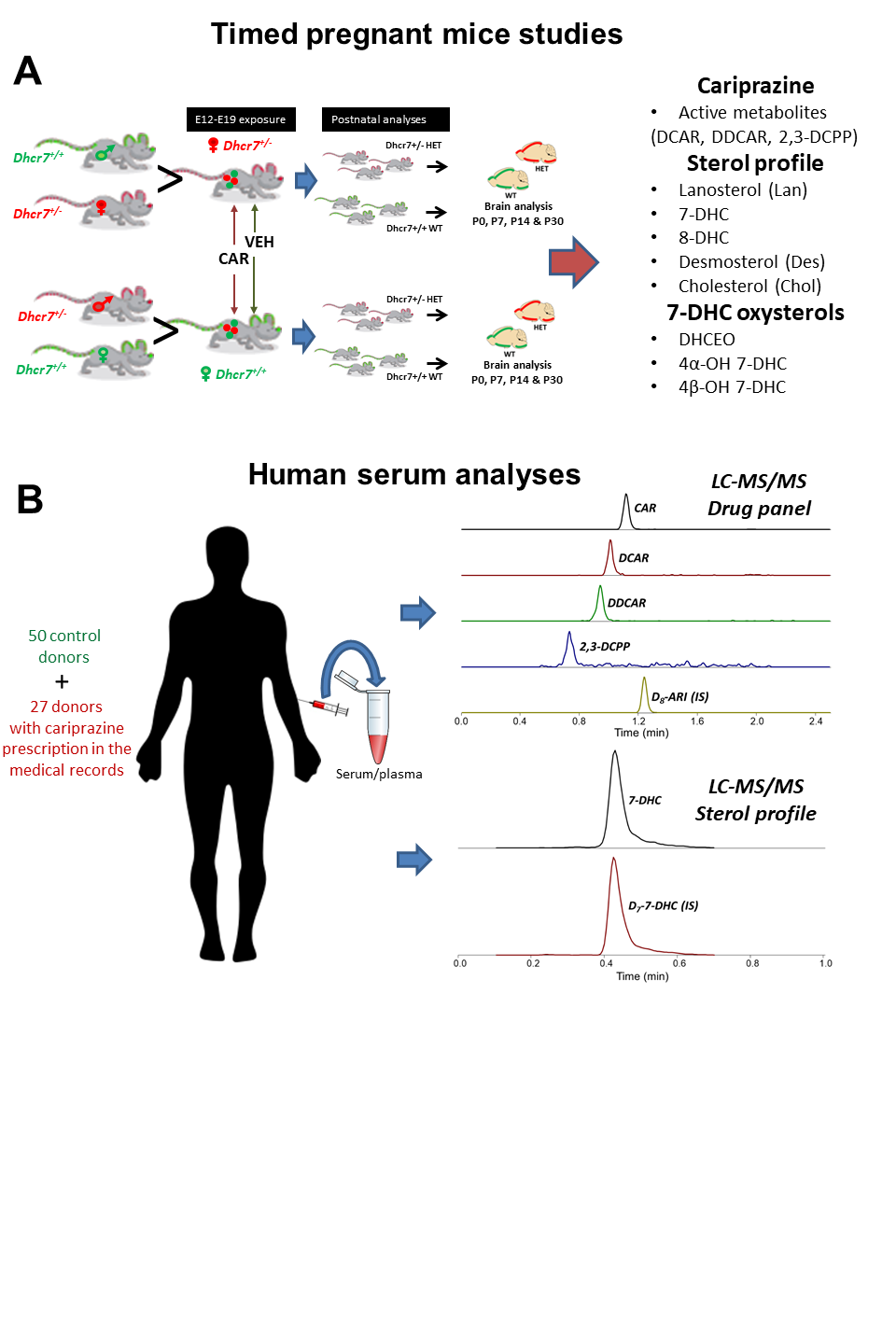
**

**Supplemental Figure 2. Experimental design. (A)** Timed pregnant *Dhcr7^+/+^* and *Dhcr7^+/-^* females were injected daily with either saline (vehicle) or 0.2 mg/kg CAR from E12 through E19 and the offspring analyzed postnatally. **(B)** Cariprazine + metabolite panel and sterol profile analyses of human serum/plasma from control individuals and patients with CAR prescription in the medical records.

**Supplemental Figure 3**


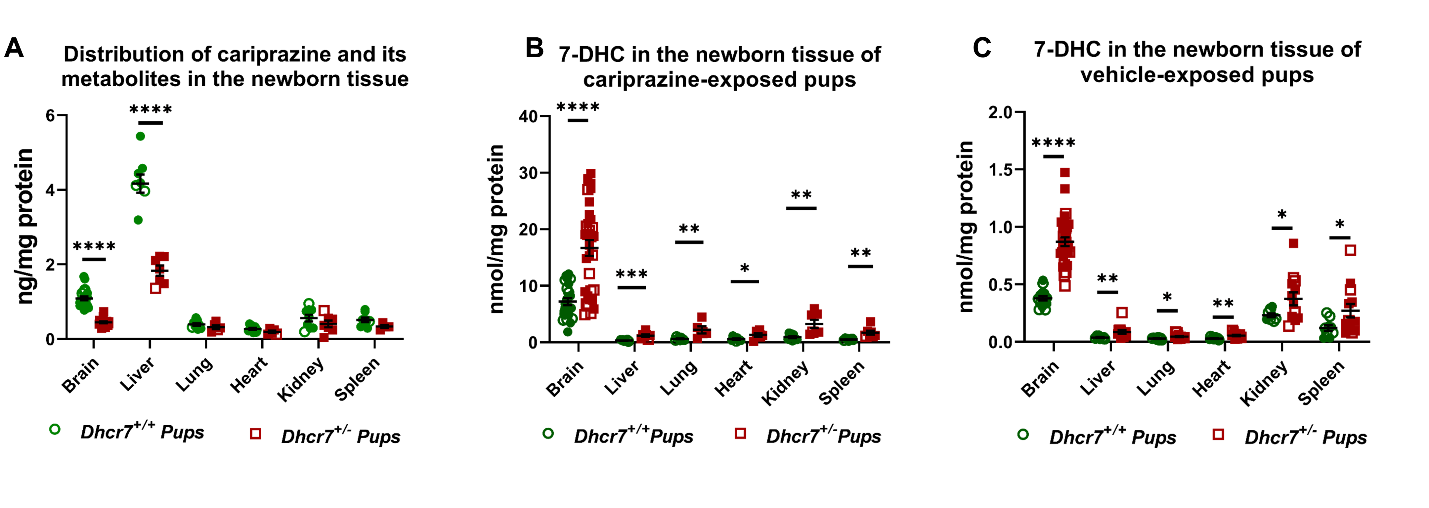


**Supplemental Figure 3. Maternal CAR exposure has a systemic effect on offspring, with the most pronounced 7-DHC elevation in brain tissue.** This figure is a different representation of data presented in **Figure 1**, denoting individual values and sex of offspring. Each symbol corresponds to a single pup brain; filled and opened symbols correspond to males and females, respectively. Scales on the y-axes are slightly different from those shown in **Figure 1** due to the variability of the individual data points.

**Supplemental Figure 4**

***
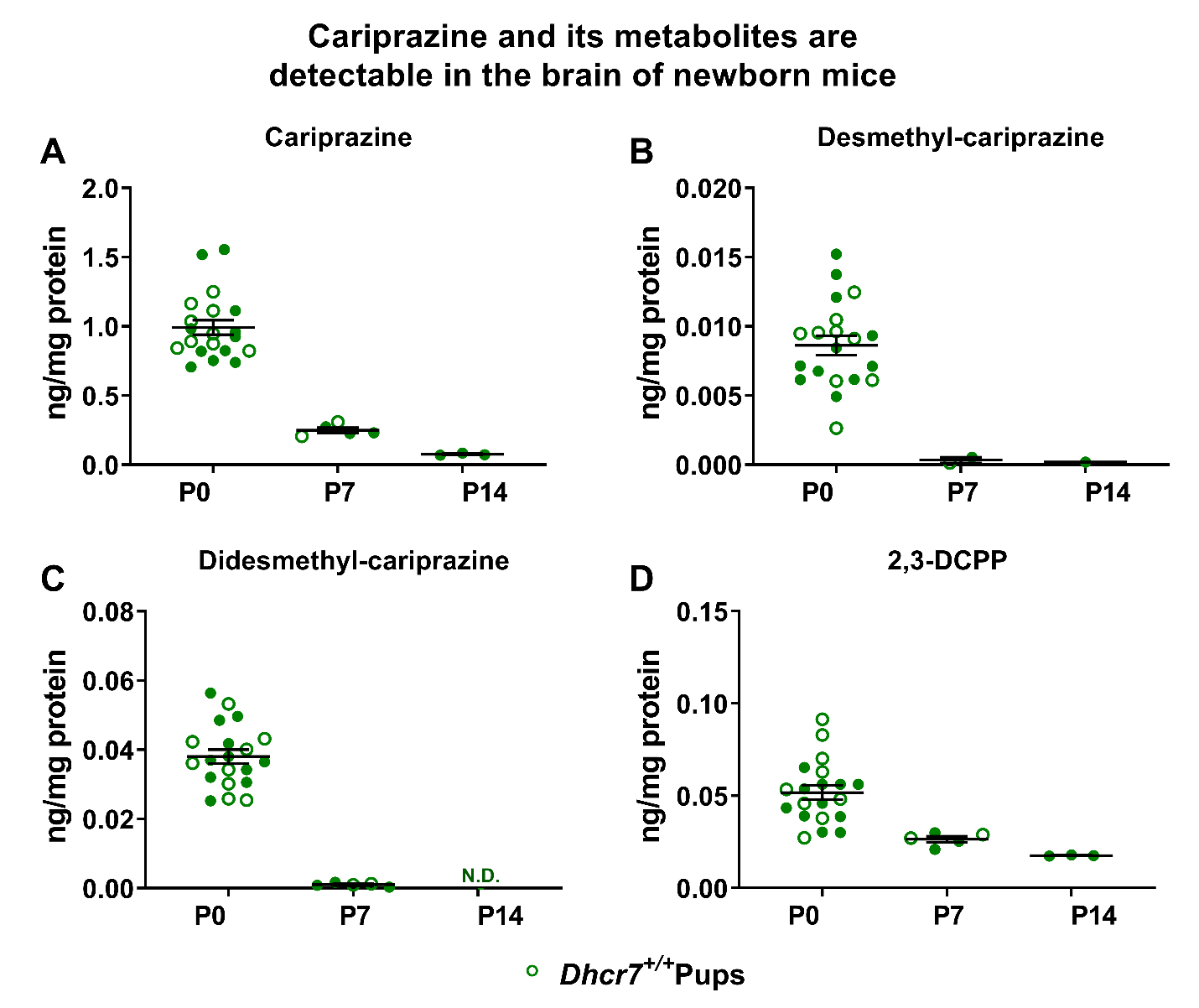
***

**Supplemental Figure 4. CAR and its active metabolites are detectable in the brain of maternally exposed offspring up to 14 days after birth.** CAR **(A)**, DCAR **(B)**, DDCAR **(C)** and 2,3-DCPP **(D)** were quantified by LC-MS/MS in the brain of WT pups exposed to the drug *in utero*. Vehicle-treated control animals are not denoted in the figure, as they had no detectable CAR or metabolite levels. N.D. = not detected. Each symbol corresponds to an individual pup brain; filled and opened symbols denote males and females, respectively; bars correspond to the mean ± SEM. CAR **(A)** is also depicted in **Figure 2A**. The chemical structures for cariprazine and its metabolites are depicted in **Supplemental Figure 1**.

**Supplemental Table 1.** Human serum samples

|  | Control | Control | Cariprazine | Cariprazine |
| --- | --- | --- | --- | --- |
| Sex | Female | Male | Female | Male |
| n | 29 | 21 | 5 | 2 |
| Age range | 25 - 50 | 29 - 44 | 26 - 49 | 32 - 40 |
| Average age | 40.3 | 38 | 41 | 36 |

*T93M and Dhcr7^Δ3–5^ mice*

T93M mice were obtained from FD Porter (1). These mice have a mutation in both *Dhcr7* alleles at c.278C>T, a mutation equivalent to the human T93M missense mutation. The T93M mutation is the most common missense mutation described in patients with SLOS (1, 2). *Dhcr7^Δ3–5/+^* was obtained from FD Porter (3). *Dhcr7^Δ3–5/+^* carries a null disruption of *Dhcr7* incorporating a deletion of coding exons 3, 4 and 5 (Δ3–5) (3). Homozygous *Dhcr7^Δ3–5/Δ3–5^* mice do not survive past birth. Compound heterozygous mice (*Dhcr7^Δ3–5/T93M^*) were obtained by breeding *Dhcr7^T93M/T93M^* with *Dhcr7^Δ3–5/+^* (4). Eight P0 *Dhcr7^Δ3–5/T93M^* and nine *Dhcr7^+/T93M^* were used for oxysterol analysis.

*Cariprazine injections in mice*

Adult male and female B6.129P2(Cg)-*Dhcr7^tm1Gst^*/J stock # 007453 mice were purchased from Jackson Laboratories. Mice homozygous for the *Dhcr7^Ex8^* allele lack the exon 8 coding sequence and flanking splice acceptor site of the targeted gene, resulting in the truncated DHCR7 mutation most frequently observed in SLOS patients (IVS8-1G>C). Homozygous mice die shortly after birth. Heterozygous *Dhcr7^+/-^* mice are well, fertile, and indistinguishable from control, wild type mice. Mice were maintained by breeding within colony and refreshing twice a year with stock 000664 mice from Jackson Laboratories. Mice were housed under a 12 h light-dark cycle at constant temperature (25°C) and humidity with *ad libitum* access to food (Teklad LM-485 Mouse/Rat Irradiated Diet 7912) and water in Comparative Medicine at the UNMC, Omaha, NE. The time-pregnant female mice received i/p injections of vehicle (VEH) or CAR (0.2 mg/kg) from E12 to E19. Eighteen WT and thirteen *Dhcr7^+/-^* mothers were used in our study. Half of each genotype group was injected with VEH and the other half with CAR. The mouse colony was monitored three times a day and all newborn pups (P0) were collected for dissection shortly after birth. Pups from a separate cohort of injected mice were also collected for tissue dissection at P7, P14, P21 and P30. Adult female mice were also sacrificed at the same time as pups. Frozen brain tissue samples were sonicated in ice-cold PBS containing butylated hydroxytoluene (BHT) and triphenylphosphine (PPh_3_). The aliquots of homogenized tissue were used for sterol extraction and protein measurements. The protein was measured using BCA assay (Pierce). All procedures were performed in accordance with the Guide for the Humane Use and Care of Laboratory Animals. The use of mice in this study was approved by the Institutional Animal Care and Use Committee of UNMC.

*Sterol analysis*

Sterols were extracted and derivatized with PTAD as described previously (5) and placed in an Acquity UPLC system equipped with ANSI-compliant well plate holder coupled to a Thermo Scientific TSQ Quantis mass spectrometer equipped with an APCI source. Then 5 μL was injected onto the column (Phenomenex Luna Omega C18, 1.6 μm, 100 Å, 2.1 mm × 50 mm) with 100% MeOH (0.1% v/v acetic acid) mobile phase for 1.0 min runtime at a flow rate of 500 μL/min. Natural sterols were analyzed by selective reaction monitoring (SRM) using the following transitions: Chol 369 → 369, 7-DHC 560 → 365, 8-DHC 558 → 363, desmosterol 592 → 560, lanosterol 634 → 602, with retention times of 0.7, 0.4, 0.3 and 0.3 min, respectively. SRMs for the internal standards were set to: d_7_-Chol 376 → 376, d_7_-7-DHC 567 → 372, d_7_-8-DHC 565 → 370, ^13^C_3_-desmosterol 595 → 563, ^13^C_3_-lanosterol 637 → 605. Final sterol numbers are reported as nmol/mg of protein.

*References*

1. Correa-Cerro LS, Wassif CA, Kratz L, Miller GF, Munasinghe JP, Grinberg A, et al. Development and characterization of a hypomorphic Smith-Lemli-Opitz syndrome mouse model and efficacy of simvastatin therapy. Hum Mol Genet. 2006;15:839-51.

2. Correa-Cerro LS, Porter FD. 3beta-hydroxysterol Delta7-reductase and the Smith-Lemli-Opitz syndrome. Mol Genet Metab. 2005;84:112-26.

3. Wassif CA, Zhu P, Kratz L, Krakowiak PA, Battaile KP, Weight FF, et al. Biochemical, phenotypic and neurophysiological characterization of a genetic mouse model of RSH/Smith--Lemli--Opitz syndrome. Hum Mol Genet. 2001;10:555-64.

4. Sharif NF, Korade Z, Porter NA, Harrison FE. Oxidative stress, serotonergic changes and decreased ultrasonic vocalizations in a mouse model of Smith-Lemli-Opitz syndrome. Genes Brain Behav. 2017;16:619-26.

5. Genaro-Mattos TC, Tallman KA, Allen LB, Anderson A, Mirnics K, Korade Z, et al. Dichlorophenyl piperazines, including a recently-approved atypical antipsychotic, are potent inhibitors of DHCR7, the last enzyme in cholesterol biosynthesis. Toxicol Appl Pharmacol. 2018;349:21-28.
